# FlexDM: Simple, parallel and fault-tolerant data mining using WEKA

**DOI:** 10.1186/s13029-015-0045-3

**Published:** 2015-11-17

**Authors:** Madison Flannery, David M. Budden, Alexandre Mendes

**Affiliations:** Systems Biology Laboratory, University of Melbourne, Parkville, 3010 VIC Australia; School of Electrical Engineering and Computer Science, The University of Newcastle, Callaghan, 2308 NSW Australia

**Keywords:** Data mining, Machine learning, XML, Java, WEKA

## Abstract

**Background:**

With the continued exponential growth in data volume, large-scale data mining and machine learning experiments have become a necessity for many researchers without programming or statistics backgrounds. WEKA (Waikato Environment for Knowledge Analysis) is a gold standard framework that facilitates and simplifies this task by allowing specification of algorithms, hyper-parameters and test strategies from a streamlined Experimenter GUI. Despite its popularity, the WEKA Experimenter exhibits several limitations that we address in our new FlexDM software.

**Results:**

FlexDM addresses four fundamental limitations with the WEKA Experimenter: reliance on a verbose and difficult-to-modify XML schema; inability to meta-optimise experiments over a large number of algorithm hyper-parameters; inability to recover from software or hardware failure during a large experiment; and failing to leverage modern multicore processor architectures. Direct comparisons between the FlexDM and default WEKA XML schemas demonstrate a 10-fold improvement in brevity for a specification that allows finer control of experimental procedures. The stability of FlexDM has been tested on a large biological dataset (approximately 450 k attributes by 150 samples), and automatic parallelisation of tasks yields a quasi-linear reduction in execution time when distributed across multiple processor cores.

**Conclusion:**

FlexDM is a powerful and easy-to-use extension to the WEKA package, which better handles the increased volume and complexity of data that has emerged during the 20 years since WEKA’s original development. FlexDM has been tested on Windows, OSX and Linux operating systems and is provided as a pre-configured virtual reference environment for trivial usage and extensibility. This software can substantially improve the productivity of any research group conducting large-scale data mining or machine learning tasks, in addition to providing non-programmers with improved control over specific aspects of their data analysis pipeline via a succinct and simplified XML schema.

**Electronic supplementary material:**

The online version of this article (doi:10.1186/s13029-015-0045-3) contains supplementary material, which is available to authorized users.

## Background

Large-scale analysis is an integral component of modern life sciences research, and is becoming a necessary day-to-day task for many researchers lacking a strong programming or statistics background. This is epitomised by genomics and epigenetics studies (e.g. those leveraging microarray and next generation sequencing technologies), which require data for tens-of-thousands of genes to be quantified and simultaneously analysed [1–3]. Sophisticated machine learning and data mining tools have been introduced to address these requirements in the life sciences and other areas of quantitative research [4]. Of these frameworks, WEKA (Waikato Environment for Knowledge Analysis) has been widely-adopted as a gold standard, having been cited more than 8000 times in academic literature [5].

Despite its proven success and widespread application, the WEKA Experimenter pipeline exhibits a number of limitations that make it both a) difficult to apply to non-trivial data mining challenges in modern research, and b) remain robust and reliable against the exponential growth of data volume in the two decades since its original development [6]. Although other third-party extensions and plug-ins have been developed to address these limitations, these have been fragmentary solutions to a subset of the underlying issues [7, 8]. The following Section expands upon four major and inter-related limitations in WEKA and how these have been addressed in our new FlexDM software.

## Implementation

FlexDM addresses four fundamental limitations identified for machine learning/data mining experiments using the WEKA Experimenter and GUI (the most common approach for non-experts, as more advanced features are unintuitive and overall poorly documented). These limitations and their FlexDM solutions are described below. It is important to note that FlexDM-generated result summaries are fully compatible with advanced WEKA features including the Analyse tab, which allows for statistical post-processing and visualisation of results.

### Reliance on WEKA experimenter GUI

**Fig. 1:**
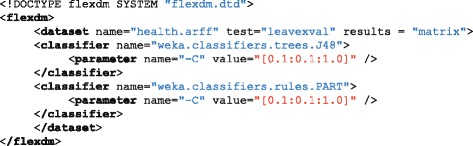
Example XML input file for FlexDM. An example FlexDM XML input file for the execution of 20 experiments, which simultaneously (and in parallel) meta-optimises the ‘confidence factor’ (c) hyper-parameter for two classification algorithms (J48 and PART). Leave-one-out cross-validation is specified as the evaluation strategy. The WEKA Experimenter equivalent is 10-fold longer and provided in Additional file 1

Although WEKA is built on-top of XML-based experiment specifications, this XML schema is highly-unreadable and clearly designed to be written exclusively from the WEKA GUI. The FlexDM XML scheme improves human readability and brevity while also providing extended functionality, as demonstrated in Fig. 1. This new schema is sufficiently readable that non-programmers are able to create their own or modify the provided example to their individual needs.

### Specification and optimisation of hyper-parameters

It is often unclear what combination of method hyper-parameters will yield the most insight from an experimental data-set, posing a need to evaluate many combinations of potential value assignments. WEKA provides only limited support for these feature through its CVParameterSelection and GridSearch functions, which are able to meta-optimise over 1-or-2 parameters respectively. FlexDM allows for numeric ranges to be specified for any number of combination of method hyper-parameters. Ranges may be represented in two forms: a set of categorical or numerical values; or a numeric interval with user-specified start, step and end values. FlexDM will automatically evaluate all combinations of parameter values and save the results as individual output files. The user can also specify their desired evaluation procedure (e.g. leave-one-out, k-fold cross-validation or test/training split).

Optimising over several hyper-parameters inherently worsens the computational time complexity of the experiment, necessitating improved fault-tolerance and the ability to parallelise tasks to leverage modern processor architectures. These features are addressed below.

### Robustness and fault-tolerance

The WEKA Experimenter runs all experiments in series and does not generate a results file until the final experiment has concluded. Many large machine learning experiments (e.g. in the life sciences [1–3]) can take hours-or-days to conclude, particularly when meta-optimising over many combinations of hyper-parameters. Any software or hardware fault during this time will cause all intermediate results to be lost. By contrast, FlexDM automatically generates individual results files upon conclusion of each experiment, and provides functionality to allow a series of experiments to automatically resume from the most recent to successfully complete.

### Asynchronous parallel processing

WEKA does not provide an intuitive means of utilising modern multi-core processing architectures, instead running a series of experiments on a single process and thread. FlexDM leverages the independent nature of individual experiments by introducing asynchronous and parallel processing. Similar features have been introduced in third-party packages including Weka-Parallel [7] and Grid-enabled Weka [8], but without the aforementioned FlexDM features necessary to maximise the value of this functionality.

## Results

This results section is separated into two parts: a practical example of the improved FlexDM XML schema when compared to its WEKA Experimenter equivalent; and an empirical analysis of the time taken to perform a large data mining task when distributed across multiple CPUs.

Figure 1 illustrates an example FlexDM XML input file for the execution of 20 experiments, which simultaneously (and in parallel) meta-optimises the ‘confidence factor’ (c) hyper-parameter for two classification algorithms (J48 and PART). Leave-one-out cross-validation was employed as the evaluation strategy, although k-fold cross-validation or percentage split could have been selected as appropriate for larger data-sets. FlexDM will load the XML file and specified data-set, asynchronously execute each experiment and summarise the results for each in individual files. FlexDM also creates a summary file reporting the overall experimental outcomes.

This easy-to-read XML specification takes only 11 lines to define a non-trivial series of experiments and hyper-parameter meta-optimisation tasks. By contrast, the equivalent WEKA Experimenter-interpreted XML specification requires 10-fold as many lines and is difficult to modify without reliance upon the GUI. These XML specifications are compared in Additional file 1.

**Fig. 2:**
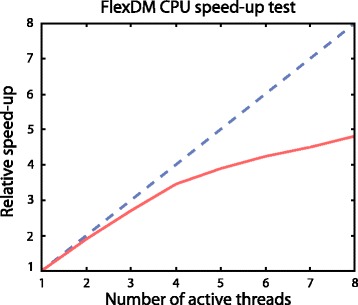
FlexDM parallel processing speed-up. Relative execution time for a FlexDM-enhanced WEKA experiment as a function of the number of allocated CPU cores (a user-specified parameter which defaults to the number of available cores minus 1). This test was completed on a desktop PC with a quad-core Intel i7 processor. A near-linear speed-up is evident for *n* = 1:4 cores, with divergence due to background system tasks necessary within the host OS environment. For *n* = 5:8 cores, the i7 processor’s hyper-threading capability is leveraged for further quasi-linear speed-up, resulting in an overall 5-fold reduction in execution time when distributed across 8 logical CPU cores

Figure 2 illustrates the relative execution time for a FlexDM-enhanced WEKA experiment as a function of the number of allocated CPU cores (a user-specified parameter which defaults to the number of available cores minus 1). This test was completed on a desktop PC with a quad-core Intel i7 processor. A near-linear speed-up is evident for *n* = 1:4 cores, with divergence due to background system tasks necessary within the host OS environment. For *n* = 5:8 cores, the i7 processor’s hyper-threading capability is leveraged for further quasi-linear speed-up, resulting in an overall 5-fold reduction in execution time when distributed across 8 logical CPU cores.

## Conclusions

FlexDM enables flexible, fault tolerant and computationally efficient data mining using WEKA. This framework has addressed four fundamental and interrelated limitations inherent to the standard WEKA Experimenter and GUI pipeline, while providing a novel XML-based specification of data mining experiments that are expressive, succinct and easily understood/extended by non-programmers. These improvements are necessary in the context of modern life sciences research, where the volume of data continues to increase at an exponential rate, and researchers without programming or statistics backgrounds are increasingly required to perform non-trivial data mining and machine learning experiments.

As we encourage other researchers to explore and adopt our software, FlexDM is implemented using exclusively open-source software and made available as a pre-configured virtual reference environment, using the approach recently described by Hurley et al*.* [9]. A comprehensive FlexDM user guide, including reference environment instructions and examples/templates of XML experiment specifications, are available online at http://madiflannery.github.io/FlexDM/

## Availability and requirements

Project Name: FlexDM

Project home page: http://madiflannery.github.io/FlexDM/

Operating system(s): Platform independent

Programming language: Java

Other requirements: Java 1.7 or higher

License: GPLv2

## Additional file

Additional file 1:
**Comparison of FlexDM and WEKA Experimenter XML specifications of equivalent experiments.** (PDF 49 kb)
