# Supplementary material for: FlexDM: Simple, parallel and fault-tolerant data mining using WEKA
Source: Source Code Biol Med. 2015 Nov 17;10:13. doi: 10.1186/s13029-015-0045-3 (PMC4647584; doi:10.1186/s13029-015-0045-3)
Supplement: Additional file 1: — Comparison of FlexDM and WEKA Experimenter XML specifications of equivalent experiments. (PDF 49 kb) [file 13029_2015_45_MOESM1_ESM.pdf]

```
<!DOCTYPE flexdm SYSTEM "flexdm.dtd">
<flexdm>
  <dataset name="health.arff" test="leavexval" results = "matrix">
    <classifier name="weka.classifiers.trees.J48">
      <parameter name="-C" value="[0.1:0.1:1.0]" />
    </classifier>
    <classifier name="weka.classifiers.rules.PART">
      <parameter name="-C" value="[0.1:0.1:1.0]" />
    </classifier>
  </dataset>
</flexdm>
```

```xml
<?xml version="1.0" encoding="utf-8"?>

<!DOCTYPE object
[
   <!ELEMENT object (#PCDATA|object)*>
   <!ATTLIST object name      CDATA #REQUIRED>
   <!ATTLIST object class     CDATA #REQUIRED>
   <!ATTLIST object primitive CDATA "no">
   <!ATTLIST object array     CDATA "no">   <!-- the dimensions of the array; no=0, yes=1 -->
   <!ATTLIST object null      CDATA "no">
   <!ATTLIST object version   CDATA "3.6.3">
]
>

<object class="weka.experiment.Experiment" name="__root__" version="3.6.3">
   <object array="yes" class="weka.classifiers.Classifier" name="propertyArray">
      <object class="weka.classifiers.meta.CVParameterSelection" name="0">
         <object class="boolean" name="debug" primitive="yes">false</object>
         <object array="yes" class="java.lang.String" name="options">
            <object class="java.lang.String" name="0">-P</object>
            <object class="java.lang.String" name="1">C 0.1 1.0 10.0</object>
            <object class="java.lang.String" name="2">-X</object>
            <object class="java.lang.String" name="3">10</object>
            <object class="java.lang.String" name="4">-S</object>
            <object class="java.lang.String" name="5">1</object>
            <object class="java.lang.String" name="6">-W</object>
            <object class="java.lang.String" name="7">weka.classifiers.trees.J48</object>
            <object class="java.lang.String" name="8">--</object>
            <object class="java.lang.String" name="9">-C</object>
            <object class="java.lang.String" name="10">0.25</object>
            <object class="java.lang.String" name="11">-M</object>
            <object class="java.lang.String" name="12">2</object>
            <object class="java.lang.String" name="13"/>
            <object class="java.lang.String" name="14"/>
            <object class="java.lang.String" name="15"/>
            <object class="java.lang.String" name="16"/>
            <object class="java.lang.String" name="17"/>
            <object class="java.lang.String" name="18"/>
            <object class="java.lang.String" name="19"/>
            <object class="java.lang.String" name="20"/>
            <object class="java.lang.String" name="21"/>
            <object class="java.lang.String" name="22"/>
         </object>
      </object>
      <object class="weka.classifiers.meta.CVParameterSelection" name="1">
         <object class="boolean" name="debug" primitive="yes">false</object>
         <object array="yes" class="java.lang.String" name="options">
            <object class="java.lang.String" name="0">-P</object>
            <object class="java.lang.String" name="1">C 0.1 1.0 10.0</object>
            <object class="java.lang.String" name="2">-X</object>
            <object class="java.lang.String" name="3">10</object>
            <object class="java.lang.String" name="4">-S</object>
            <object class="java.lang.String" name="5">1</object>
            <object class="java.lang.String" name="6">-W</object>
            <object class="java.lang.String" name="7">weka.classifiers.rules.PART</object>
            <object class="java.lang.String" name="8">--</object>
            <object class="java.lang.String" name="9">-M</object>
            <object class="java.lang.String" name="10">2</object>
            <object class="java.lang.String" name="11">-C</object>
            <object class="java.lang.String" name="12">0.25</object>
            <object class="java.lang.String" name="13">-Q</object>
            <object class="java.lang.String" name="14">1</object>
            <object class="java.lang.String" name="15"/>
            <object class="java.lang.String" name="16"/>
            <object class="java.lang.String" name="17"/>
            <object class="java.lang.String" name="18"/>
            <object class="java.lang.String" name="19"/>
         </object>
      </object>
   </object>
   <object class="javax.swing.DefaultListModel" name="datasets">
      <object class="java.io.File" name="0">C:\Users\Alexandre\Desktop\health.arff</object>
   </object>
   <object class="int" name="runUpper" primitive="yes">1</object>
   <object class="weka.experiment.CrossValidationResultProducer" name="resultProducer">
      <object array="yes" class="java.lang.String" name="options">
         <object class="java.lang.String" name="0">-X</object>
         <object class="java.lang.String" name="1">930</object>
         <object class="java.lang.String" name="2">-O</object>
         <object class="java.lang.String" name="3">splitEvalutorOut.zip</object>
         <object class="java.lang.String" name="4">-W</object>
         <object class="java.lang.String" name="5">weka.experiment.ClassifierSplitEvaluator</object>
         <object class="java.lang.String" name="6">--</object>
         <object class="java.lang.String" name="7">-W</object>
         <object class="java.lang.String" name="8">weka.classifiers.rules.ZeroR</object>
         <object class="java.lang.String" name="9">-I</object>
         <object class="java.lang.String" name="10">0</object>
         <object class="java.lang.String" name="11">-C</object>
         <object class="java.lang.String" name="12">1</object>
         <object class="java.lang.String" name="13">--</object>
         <object class="java.lang.String" name="14"/>
         <object class="java.lang.String" name="15"/>
      </object>
   </object>
   <object class="boolean" name="usePropertyIterator" primitive="yes">true</object>
   <object array="yes" class="weka.experiment.PropertyNode" name="propertyPath">
      <object array="no" class="weka.experiment.PropertyNode" name="0" primitive="no">
         <object class="weka.experiment.ClassifierSplitEvaluator" name="value">
            <object array="yes" class="java.lang.String" name="options">
               <object class="java.lang.String" name="0">-W</object>
               <object class="java.lang.String" name="1">weka.classifiers.rules.ZeroR</object>
               <object class="java.lang.String" name="2">-I</object>
               <object class="java.lang.String" name="3">0</object>
               <object class="java.lang.String" name="4">-C</object>
               <object class="java.lang.String" name="5">1</object>
               <object class="java.lang.String" name="6">--</object>
               <object class="java.lang.String" name="7"/>
            </object>
         </object>
         <object class="java.lang.String" name="parentClass">weka.experiment.CrossValidationResultProducer</object>
         <object class="java.lang.String" name="property">splitEvaluator</object>
      </object>
      <object array="no" class="weka.experiment.PropertyNode" name="1" primitive="no">
         <object class="weka.classifiers.rules.ZeroR" name="value">
            <object class="boolean" name="debug" primitive="yes">false</object>
            <object array="yes" class="java.lang.String" name="options"/>
         </object>
         <object class="java.lang.String" name="parentClass">weka.experiment.ClassifierSplitEvaluator</object>
         <object class="java.lang.String" name="property">classifier</object>
      </object>
   </object>
   <object class="java.lang.String" name="notes"/>
   <object class="weka.experiment.InstancesResultListener" name="resultListener">
      <object array="yes" class="java.lang.String" name="options">
         <object class="java.lang.String" name="0">-O</object>
         <object class="java.lang.String" name="1">weka_experiment2432505407983808771.arff</object>
      </object>
      <object class="java.io.File" name="outputFile">C:\Users\ALEXAN~1\AppData\Local\Temp\weka_experiment2432505407983808771.arff</object>
   </object>
   <object class="boolean" name="advanceDataSetFirst" primitive="yes">true</object>
   <object class="int" name="runLower" primitive="yes">1</object>
   <object class="java.lang.Boolean" name="classFirst">false</object>
</object>
```
